# Characterization of miRNAs in Embryonic, Larval, and Adult Lumpfish Provides a Reference miRNAome for *Cyclopterus lumpus*

**DOI:** 10.3390/biology11010130

**Published:** 2022-01-13

**Authors:** Setu Chakraborty, Nardos T. Woldemariam, Tina Visnovska, Matthew L. Rise, Danny Boyce, Javier Santander, Rune Andreassen

**Affiliations:** 1 Marine Microbial Pathogenesis and Vaccinology Laboratory, Department of Ocean Sciences, Memorial University of Newfoundland, 0 Marine Lab Rd, St. John’s, NL A1C 5S7, Canada; schakraborty@mun.ca; 2 Department of Life Sciences and Health, Faculty of Health Sciences, OsloMet–Oslo Metropolitan University, Pilestredet 50, N-0130 Oslo, Norway; nardostwoldemariam@gmail.com; 3 Bioinformatics Core Facility, Oslo University Hospital, 0372 Oslo, Norway; tina.visnovska@rr-research.no; 4 Department of Ocean Sciences, Faculty of Sciences, Memorial University of Newfoundland, 0 Marine Lab Rd, St. John’s, NL A1C 5S7, Canada; mrise@mun.ca; 5 Dr. Joe Brown Aquatic Research Building (JBARB), Department of Ocean Sciences, Memorial University of Newfoundland, 0 Marine Lab Rd, St. John’s, NL A1C 5S7, Canada; dboyce@mun.ca

**Keywords:** conserved miRNA, high-throughput sequencing, lumpfish, novel miRNA, RT-qPCR

## Abstract

**Simple Summary:**

Lumpfish (*Cyclopterus lumpus*) is an emergent aquaculture species, and its miRNA repertoire is still unknown. miRNAs are critical post-transcriptional modulators of teleost gene expression. Therefore, a lumpfish reference miRNAome was characterized by small RNA sequencing and miRDeep analysis of samples from different organs and developmental stages. The resulting miRNAome, an essential reference for future expression analyses, consists of 443 unique mature miRNAs from 391 conserved and eight novel miRNA genes. Enrichment of specific miRNAs in particular organs and developmental stages indicates that some conserved lumpfish miRNAs regulate organ and developmental stage-specific functions reported in other teleosts.

**Abstract:**

MicroRNAs (miRNAs) are endogenous small RNA molecules involved in the post-transcriptional regulation of protein expression by binding to the mRNA of target genes. They are key regulators in teleost development, maintenance of tissue-specific functions, and immune responses. Lumpfish (*Cyclopterus lumpus*) is becoming an emergent aquaculture species as it has been utilized as a cleaner fish to biocontrol sea lice (e.g., *Lepeophtheirus salmonis*) infestation in the Atlantic Salmon (*Salmo salar*) aquaculture. The lumpfish miRNAs repertoire is unknown. This study identified and characterized miRNA encoding genes in lumpfish from three developmental stages (adult, embryos, and larvae). A total of 16 samples from six different adult lumpfish organs (spleen, liver, head kidney, brain, muscle, and gill), embryos, and larvae were individually small RNA sequenced. Altogether, 391 conserved miRNA precursor sequences (discovered in the majority of teleost fish species reported in miRbase), eight novel miRNA precursor sequences (so far only discovered in lumpfish), and 443 unique mature miRNAs were identified. Transcriptomics analysis suggested organ-specific and age-specific expression of miRNAs (e.g., miR-122-1-5p specific of the liver). Most of the miRNAs found in lumpfish are conserved in teleost and higher vertebrates, suggesting an essential and common role across teleost and higher vertebrates. This study is the first miRNA characterization of lumpfish that provides the reference miRNAome for future functional studies.

## 1. Introduction

The discovery of microRNAs (miRNAs) in 1993 in *Caenorhabditis elegans* and further identification in humans and many other animals significantly alters the longstanding dogmas that defined gene regulation [1]. These studies revealed that miRNAs were a class of small noncoding RNAs that function as guide molecules in RNA silencing machinery, often termed the RNA-induced silencing complex (RISC). RISC regulates gene expression at the messenger RNA level either by degrading mRNAs targeted by the miRNAs or preventing their translation [1,2,3]. miRNA constitute a large family of post-transcriptional regulators with ~22 nucleotides in length and present in animals, plants, and some viruses [3,4]. Functional studies indicate that miRNAs have diverse expression patterns and regulate almost every cellular process, including developmental, physiological, and pathophysiological processes [3,5,6].

miRNA biogenesis involves multiple steps; first, miRNAs are processed from primary molecules (pri-miRNAs), which are transcribed by RNA-specific endoribonuclease (Drosha) and processed into an ~70-nucleotide pre-miRNA in the nucleus [2,3,6,7,8,9]. Pre-miRNAs are then transported to the cytoplasm for further processing by the enzyme Dicer to an ~22-bp miRNA/miRNA duplex [2,3,6,7,8,9]. The miRNA duplex is loaded into the RISC. Only one of the mature miRNAs (guide miRNA) is incorporated in RISC, and the other is degraded (passenger miRNA). The guide miRNA directs the RISC to target mRNAs, where the mature miRNA usually binds in the 3ʹ untranslated region (UTR) of the target mRNAs [2,3,6,7,8,9]. 

Teleosts are an essential component of aquatic ecosystems and a primary source of proteins for human and animal consumption worldwide. Teleosts are one of the most diverse vertebrates on the earth [10]. The exploration of the role of miRNA in teleost development, organogenesis, tissue differentiation, growth, regeneration, reproduction, endocrine system, and responses to environmental stimuli, as well as their role in the maturation of the immune system and response to infectious diseases, is still under investigation [11,12,13,14,15,16,17]. miRNA characterization is the first step in any investigation of their regulatory roles. Such characterizations have been carried out in some economically important fish species such as Atlantic salmon (*Salmo salar*), Atlantic cod (*Gadus morhua*), rainbow trout (*Oncorhynchus mykiss*), Atlantic halibut (*Hippoglossus hippoglossus*), channel catfish (*Ictalurus punctatus*), turbot (*Scophthalmus maximus*), Asian seabass (*Lates calcarifer*), olive flounder (*Paralichthys olivaceus*) [18,19,20,21,22,23,24,25,26], and fish models like zebrafish (*Danio rerio*) and three-spined stickleback (*Gasterosteus aculeatus*) [27,28]. 

Global production of farmed Atlantic salmon is estimated at just over 2.6 million tonnes in 2019. This growth was mainly driven by Norway and Chile, the two leading producing countries. The Norwegian salmon industry alone earned some NOK 19–20 billion (USD 2.1–2.2 billion) in profits before tax in 2019 (The Food and Agriculture Organization of United Nations (FAO), 2021) (https://www.fao.org/in-action/globefish/market-reports/resource-detail/en/c/1268636/) (Last accesed: 21 January 2021). Farmed salmon is the main species of the Atlantic Canadian aquaculture industry, which represents approximately 80% of Atlantic Canada’s total aquaculture value [29].

Infectious diseases are a challenge for the aquaculture industry. Globally losses due to diseases in the aquaculture industry exceed US$6 billion annually [30,31]. One of the most prominent disease challenges currently restraining Atlantic salmon aquaculture is the infestation by the parasite sea lice, specifically *Lepeophtheirus salmonis* and *Caligus* spp. [32,33,34,35,36]. Sea lice are a group of visible host-dependent ectoparasite copepods with vast reproductive potential [32,33,34,35,36]. The attached sea lice feed on salmon mucus, blood, and skin, which leads to significant physical damage and immunosuppression [32,36,37,38,39]. In addition, these effects on fish health lead to substantial economic impacts due to production losses and treatment costs [32,37,40]. The salmon industry in the North Atlantic region has adopted cleaner fish, e.g., Lumpfish (*Cyclopterus lumpus*), for biological control of sea-lice infestations [32,41,42,43,44]. However, several aspects of lumpfish biology remain unknown, including their miRNA repertoire. 

This study aimed to identify and characterize miRNA encoding genes in lumpfish by small RNAs high-throughput sequencing (HTS) followed by miRDeep2 analysis. The identification was carried out in two early developmental stages, embryos and larvae, and six organs of adult lumpfish—brain, muscle, gill, liver, spleen, and head kidney. A combination of HTS and computational analytical approaches (e.g., miRNA precursor prediction) has been successfully used for miRNA characterization in particular two early developmental stages and adult lumpfish organs. Therefore, here we provide the first reference miRNAome for lumpfish. 

## 2. Materials and Methods

### 2.1. Fish Holding

Five adult lumpfish (1100 g ± 99.5, male = 4, female = 1) were obtained from the Joe Brown Aquatic Research Building (JBARB) at the Department of Ocean Sciences (DOS), Memorial University of Newfoundland (MUN), Canada. The animals were kept in a 23,000 L tank, with flow-through (140 L min^−1^) of UV-treated seawater (6 °C), ambient photoperiod (winter–spring), and 95–110% air saturation. Biomass density was maintained at 20 kg m^−3^. The fish were fed daily using a commercial diet (Skretting—Europa 18 (6.0–9.0 mm pellet), Vancouver, BC, Canada) with a ratio of 0.25% of their body weight per day. Additionally, lumpfish embryos (300 degree days) and lumpfish larvae (one week posthatch) were obtained from the JBARB. Lumpfish egg masses were fertilized and maintained with flow-through in 5 L upwelling black nontranslucent incubators at 8–10 °C supplied with 95–110% air saturated and 5 µm UV-treated filtered flow-through seawater (spring–summer) [45]. After completing the development of the embryo, the larvae hatch are maintained at 10 °C [46].

### 2.2. Ethics Statement

The fish dissection and tissue sample collection were performed following the Canadian Council on Animal Care guidelines (https://ccac.ca/en/standards/guidelines/) (Last accessed: 10 January 2022) and approved by Memorial University of Newfoundland’s Institutional Animal Care Committee (https://www.mun.ca/research/about/acs/) (Last accessed: 10 January 2022) under the protocols #18-1-JS and #18-03-JS.

### 2.3. Sample Collection 

Fish were euthanized with 400 mg of MS222 (Syndel Laboratories, Vancouver, BC, Canada) per litre of seawater and dissected immediately after death confirmation. Tissue samples were collected from adult lumpfish brain, gill, skeletal muscle, liver, spleen, and head kidney. Tissue samples from five adult lumpfish, two pools of lumpfish embryos, and two pools of lumpfish larvae were immediately flash-frozen in liquid nitrogen (Air Liquide Canada Atlantic, St. John’s, NL, Canada) and stored at −80 °C until further processing.

### 2.4. RNA Extraction

Total RNA was extracted using the mirVana RNA isolation kit (Invitrogen, Carlsbad, CA, USA) following the manufacturer’s protocol. RNA concentration and integrity were quantified using spectrophotometry (Genova-nano, Jenway, Stone, Staffordshire, England) and 1% agarose gel electrophoresis. The total RNA concentrations of 32 samples (four adult individuals with brain and muscle samples, five adult individuals with gill, liver, spleen, and head kidney samples, and four samples from two early life stages) ranged from 100–3250 ng μL^−1^ (total volume 100 μL) (Table S1). Sixteen samples from two adult lumpfish, two embryos, and two larvae were used for high-throughput sequencing (HTS) by independent library preparation and sequencing of each sample (Table S2), whereas all the 32 samples were used for qPCR analysis. 

### 2.5. High-Throughput Sequencing (HTS) 

The library construction and small RNA sequencing were performed by the Genomics Core Facility Oslo (Oslo University Hospital, Oslo, Norway). The NEBNext Small RNA Library Prep Set for Illumina (New England Biolabs, Inc., Ipswich, MA, USA) was used to prepare the libraries according to the manufacturer’s protocol. One µg of total RNA from each sample was used as input to prepare the libraries, and a final size selection of 140–150 bp fragments using 6% polyacrylamide gel was used to enrich small RNAs. The adapter sequences (5′ AGATCGGAAGAGCACACGTCTGAACTCCAGTCAC 3′) were used in the library preparation process. Following library preparation, next-generation RNA sequencing was carried out using the Illumina Genome Analyzer IIx sequencing platform described in Woldemariam et al. [19], generating single-end reads of length 75 bp. 

### 2.6. Analysis of Sequencing Data

The sequencing of raw and processed data quality was checked before miRNA discovery analysis. FastQC v0.11.9 (http://www.bioinformatics.babraham.ac.uk/projects/fastqc/) (Last accessed: 20 November 2021) was used to check the quality of both the raw sequencing data and the data obtained after adapter removal and size filtering. The adapter removal and size filtering were carried out using cutadapt v1.8.3 [47]. Reads shorter than 18 bp and longer than 25 bp after the adapter removal were discarded. The reads passing the filtering step were converted to fasta format with fastq_to_fasta from FASTX toolkit v0.0.14 (http://hannonlab.cshl.edu/fastx_toolkit/) (Last accessed: 20 November 2021). 

**Figure 1:**
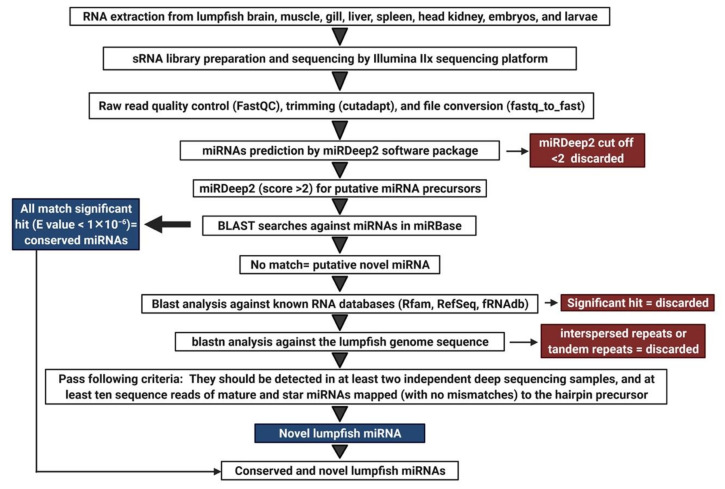
Experimental workflow used for characterization of lumpfish miRNAome.

Each of the 16 samples were analyzed independently to detect miRNAs highly expressed in particular adult organs/tissues and early developmental stages. The lumpfish reference genome [48] and bowtie v1.0.0 [49] were used for mapping the reads to the reference genome. The workflow applied to identify novel lumpfish miRNA sequences is illustrated in Figure 1.

High-quality trimmed reads were used to discover lumpfish miRNA using the miRDeep2 software package v0.0.7 (mapper and miRDeep2 analysis modules) applying default commands [50,51]. The miRDeep2 tools assign a log-odds score (the miRDeep2 score) based on an algorithm that integrates the statistics of the read positions, the frequencies of reads within hairpins, and the posterior probability that the hairpin was derived from a true miRNA gene [50]. A miRDeep2 score of ≥2 was used as a cutoff to prevent false positive detection of miRNA precursors. In addition, they were inspected regarding the following criteria: (i) reads between 5′ and 3′ end of a precursor should be aligned perfectly in a discrete manner; (ii) miRNA precursors should be detected in at least two independent deep sequencing samples, and (iii) at least ten sequence reads of mature and miRNAs mapped to the hairpin precursor [52]. We further analyzed these putative precursor sequences by BLAST searches against known precursor sequences deposited in miRBase, (http://www.mirbase.org/index.shtml) (last accessed on 22 November 2021). Any putative miRNA precursor sequence having a significant hit (E-value < 1 × 10^−6^) in the BLAST analyses was regarded as a true evolutionarily conserved lumpfish ortholog of the miRNA gene in miRBase that retrieved the best hit and annotated as the evolutionarily conserved lumpfish ortholog of the miRNA gene according to the miRbase nomenclature guidelines (clu-prefix and same number as in other teleosts) [53,54]. The putative miRNA precursor sequences that were identified by miRDeep2 and passed the additional criteria but did not show any significant match to the existing precursors in miRBase were considered putative novel miRNAs. All those sequences were further analyzed by blastn searches against RNA databases in GenBank (http://blast.ncbi.nlm.nih.gov/Blast) (Last accessed: 20 November 2021), the small RNA databases Rfam (https://rfam.xfam.org/search) (Last accessed: 20 November 2021), and the functional RNA database fRNAdb (https://dbarchive.biosciencedbc.jp/en/frnadb/desc.html) (Last accessed: 20 November 2021). Sequences that had a significant hit against these databases were considered other kinds of small RNA and discarded from the analysis. The remaining precursors were used as queries in blastn analysis against the lumpfish genome sequence. Sequences with a significant BLAST hit (E value < 1 × 10^−6^) against multiple loci (>10) in the lumpfish genome reference sequence were considered to be interspersed repeats or tandem repeats and discarded from the analysis. Sequences that passed all these filtering steps were regarded as true novel lumpfish miRNAs. A reference miRNAome of unique mature miRNA sequences (5p or 3p) for expression analysis of HTS data were generated by aligning all mature miRNAs using Sequencher software 5.3 (Gene Codes Corporation, Ann Arbor, MI, USA). The identical mature miRNAs from the same families were aligned applying strict settings, and the final reference, thus, consisted of only the unique, different mature miRNAs.

### 2.7. Disclosing Putative Differentially Expressed and Organ and Developmental Stage Enriched miRNAs 

The HTS data from 16 tissue samples were used to estimate the expression of individual miRNAs across the different organs and developmental stages. The adapters were trimmed from the raw reads, and the resulting reads were filtered based on size. The filtered reads from all 16 samples were mapped to the reference applying STAR aligner software (v.2.5.2b) [47]. The index for mapping was generated from the unique mature lumpfish miRNAs (see 2.6) with parameters genomeSAindexNbases 6. STAR aligner software (v.2.5.2b) with alignIntronMax1 and default parameters was then used for the mapping. Next, the output files of STAR mapping (BAM format) were processed further in R-Studio by using the feature Counts function from the Rsubread package (v.1.34.2) to produce count matrices [47]. The count tables were used as input in the DESeq2 R package (v.1.24.0) for differential expression analysis. Samples from an organ or developmental stage (*n* = 2) were compared to all other tissues sampled (*n* = 14). Putative differentially expressed miRNAs were defined as those with Benjamini-Hochberg adjusted *p* ≤ 0.05, log_2_ fold change threshold value of at least ≤−3.0 or ≥3.0. The miRNA abundance of the different miRNAs within a particular organ or developmental stage was estimated as the percentage of a specific miRNA out of the total based on the average of normalized read counts from duplicated samples (reads less than 20 were filtered out). Enriched miRNAs were analyzed for each organ and developmental stage. 

### 2.8. RT-qPCR

We selected eight different miRNAs that were suggested as differentially expressed in literature and enriched in one of the organs by the DESeq2 analysis for further expression analysis with qPCR. These miRNAs had previously shown similar organ-specific enrichment in other teleosts [19,20]. The RNA-seq read numbers of these eight miRNAs are provided in Table S8. Those eight miRNAs (clu-miR-135c-5p, clu-miR-9b-3p, clu-miR-133ab-3p, clu-miR-205-1-5b, clu-miR-203-3p, clu-miR-203a-5p, clu-miR-192a-5p, clu-miR-122-1-5p) were analysed by RT-qPCR to verify the DESeq2 results. All forward primer sequences used for qPCR were retrieved from the mature sequences of these miRNAs in the characterization step (methods 2.6). The primer sequences are listed in Table 1. The cDNA synthesis and qPCR were carried out applying the miScript (miScript II RT Kit and miScript SYBR Green PCR Kit) assays following the manufacturer’s instructions (Qiagen, Hilden, Germany). The qPCR reaction mixture contained 12.5 μL 2 × QuantiTect SYBR Green Master Mix, 2.5 μL 10× miScript Universal Primer, 2.5 μL of 10 μM forward miRNA-specific primer, 5 μL RNase free water, and 2.5 μL cDNA. The qPCR analysis was carried out by Mx3000p (Stratagene, Agilent Technologies, LA Jolla, CA, USA) using the following cycle, 95 °C for 15 min followed by 40 cycles of 94 °C for 15 s, 55 °C for 30 s and 70 °C for 30 s as described in Andreassen et al., 2016 [20]. The mature sequences of clu-mir-25-3p and clu-mir-17-5p were used as reference genes [20,55]. The instrument-provided ct values were applied to the LinRegPCR (v2021.1) software to calculate efficiency in all assays, and then the efficiency-adjusted Ct-values were provided [56]. The efficiency adjusted values were also used in the normalization (geomean from the two reference genes) to provide the dCt-values. The relative change in expression in each miRNA’s target organ was calculated using the comparative Ct method (ΔΔCt-method) [57]. All the comparisons were relative to the lowest expressed organ/tissue for the particular miRNA. All relative quantity (RQ) data are presented as mean ± standard error (SE). The RQ values for each target gene were subjected to a one-way ANOVA with Tukey post-tests to compare gene expression across tissues. All statistical tests were performed using GraphPad Prism 7.04 (San Diego, CA, USA) with the *p*-value threshold set at ≤0.05. The number of organ samples was four or five for each group, while the early developmental stages had two biological replicates in each group (Table S1). 

## 3. Results

### 3.1. Total RNA Extraction, Library Preparation, and Small RNA Sequencing

Total RNA extracted from 32 samples (brain, muscle, gill, liver, spleen, and head kidney from five adult fish and two samples each from larvae and embryos) showed concentrations ranging from 100 to 3250 ng/µL (Table S1) and intact 28S and 18S bands in 1% agarose gel indicated that they were of high quality. All these samples qualified for further analysis by HTS and qPCR. Small RNA libraries were successfully generated for 16 samples (twelve tissue samples from two adult fish and two samples from each early developmental stage). The HTS resulted in a total of 147,972,041 raw reads, ranging from 6.6 to 13.3 million reads per sample. After adapter trimming, there were a total of 86,054,423 reads ranging from 4.5 to 6.9 million reads per sample (Table S2). All raw HTS results were submitted to NCBI with BioProject accession number PRJNA679415. The individual SRA accession numbers are given in Table S2.

### 3.2. Characterization of Lumpfish miRNA

The processed reads from each sample were analyzed with miRDeep2 software for miRNA gene discovery (Figure 1). Subsequent BLAST homology searches of all putative miRNA precursor sequences against miRbase revealed a total of 391 miRNA genes from 104 different families that were lumpfish orthologs to evolutionarily conserved miRNAs. They were subsequently annotated as the lumpfish orthologs of these miRNAs. The miRDeep2 analysis also revealed 5p or 3p arm domination (most abundant mature miRNA from a given precursor) and the genome location of each miRNA gene. An overview of all precursor sequences along with their corresponding 5p and 3p mature sequences is given for all evolutionarily conserved miRNA genes in Table S3. 

A total of 98 precursors identified by miRDeep2 did not show significant matches in the homology analyses against miRBase. These were considered as putative novel miRNA precursor sequences. They were further analyzed by blastn searches against RNA databases in GenBank, small RNA databases Rfam, functional RNA database fRNAdb, and lumpfish genome sequence (GenBank Accession: PRJNA625538). Sequences that had a significant hit against these databases were discarded from the analysis, as described in the methods section. Following this filtering process, eight precursor and corresponding mature sequences showed characteristics expected from true miRNAs. These eight miRNA precursor sequences are likely to represent novel lumpfish miRNAs, and all these novel miRNA genes along with their corresponding 5p and 3p mature sequences, the observed arm dominance of mature sequences, and the genome location of each miRNA gene are given in the last part of Table S3. Finally, the mature miRNAs were aligned using Sequencher software to identify all unique mature miRNAs (many mature miRNAs from the same families were identical). There were 443 unique mature miRNAs. These unique miRNAs representing the lumpfish miRNAome are given in Supplementary File S4.

### 3.3. Abundance of miRNAs within Organs and Developmental Stages

We determined the diversity of miRNAs within the lumpfish tissues/organs and developmental stages based on the normalized read counts. The normalized read counts for all samples are shown in Table S5, while the average normalized read counts for each tissue/organ or developmental stage are shown in Table S6. 

**Figure 2:**
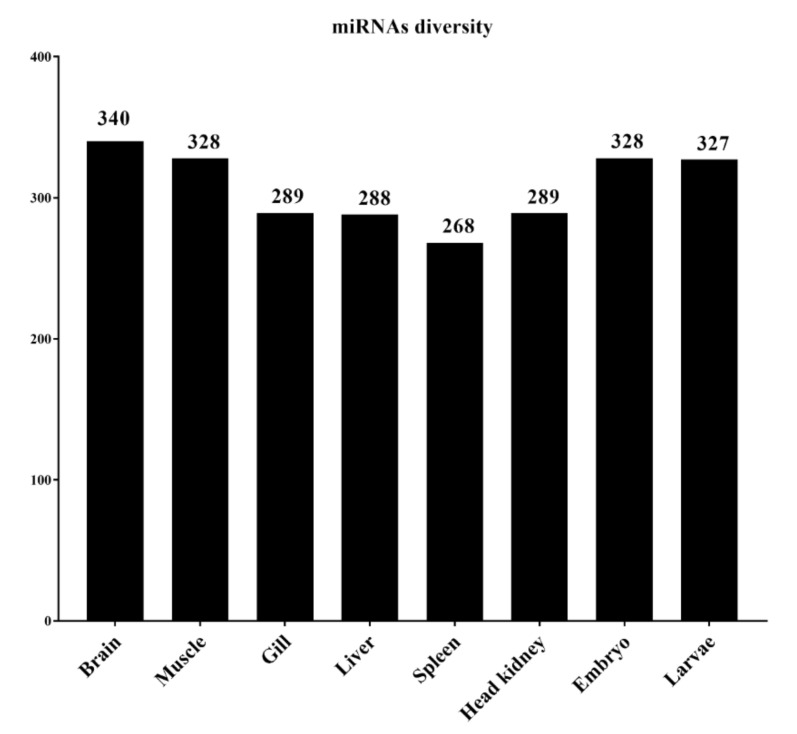
miRNA diversity in lumpfish tissue/organs and early developmental stages.

Our results show the presence of 340 unique mature miRNAs in the lumpfish brain, 328 in muscle, 289 in gill, 288 in the liver, 268 in the spleen, 289 in the head kidney, 328 in embryos, and 327 in larvae (Figure 2). Two hundred forty-one mature miRNAs were expressed in all six organs of adult lumpfish, 324 mature miRNAs were expressed commonly in embryos and larvae, and 223 mature miRNAs were expressed across all three developmental stages. All the miRNAs expressed in the early life stages, such as embryos and larvae, were also expressed in at least one organ of adult fish. The exceptions were clu-miR-19a-2-5p, which was only expressed in embryos, and clu-miR-137-1-5p, only in larvae.

**Figure 3:**
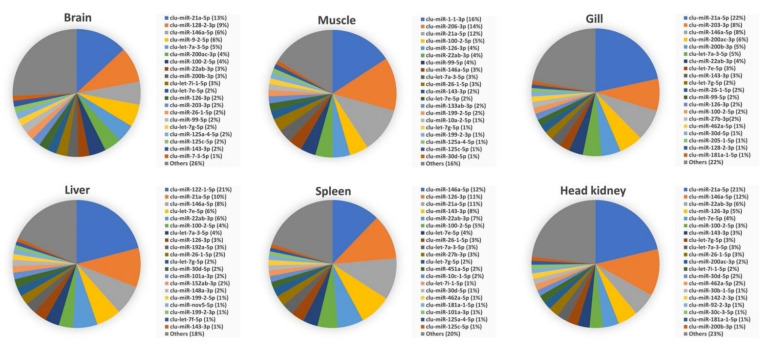
Twenty most abundant miRNAs in lumpfish brain, muscle, gill, liver, spleen, and head kidney.

**Figure 4:**
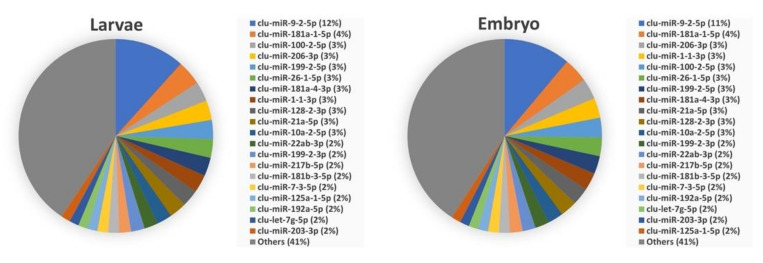
Twenty most abundant miRNAs in lumpfish embryos and larvae.

The abundance of most common mature miRNAs within each organ and developmental stage is shown in Figure 3 and Figure 4, respectively. These figures show the distribution of the top 20 enriched mature miRNAs within each of the six organs and in the two early developmental stages. The abundances for all miRNAs within each of the organs and early developmental stages are shown in Table S6. Five of the top 20 enriched mature miRNAs, clu-miR-21a-5p, clu-miR-22ab-3p, clu-miR-26-1-5p, clu-miR-100-2-5p, and clu-let-7g-5p were highly abundant within all organ and early developmental stages. While the five mature miRNAs clu-miR-146a-5p, clu-let-7a-3-5p, clu-miR-126-3p, clu-let-7e-5p, and clu-miR-143-3p were highly abundant miRNAs within all six organs of adult lumpfish, but not among the highly expressed miRNAs within lumpfish embryos and larvae (Figure 3 and Figure 4). Additionally, several miRNAs were highly abundant within one of the tissue/organs from adult fish compared to others. For example, Clu-miR-122-1-5p, clu-miR-192a-5p, clu-miR-152ab-3p, and one novel miRNA (clu-miR-nov-5-5p) were also among the top 20 most abundant miRNAs in the liver, but with much lower abundance when comparing expression of miRNAs within other organs. Likewise, clu-miR-1-1-3p, clu-miR-206-3p, and clu-miR-133ab-3p were abundant only in muscle, clu-miR-451a-5p only in spleen, clu-miR-142-2-3p only in head kidney, and clu-miR-9-2-5p and clu-miR-7-3-5p only in brain (Figure 3, Table S6). Two miRNAs, clu-miR-217b-5p and clu-miR-181b-3-5p, were common in the two early developmental stages while having relatively low expression within adult organs. In addition, there were some miRNAs common in one organ and early developmental stage. These were clu-miR-9-2-5p and clu-miR-7-3-5p (brain and early developmental stages), clu-miR-1-1-3p and clu-miR-206-3p (brain and early developmental stages) and clu-miR-192a-5p (liver and early developmental stages).

### 3.4. Comparison of Mature miRNA Expression between Organs and Early Developmental Stages

To further explore whether some miRNAs (any of the miRNAs, not only top common ones) were differentially expressed between adult organs or early developmental stages, we carried out expression analysis of the HTS data and additional RT-qPCR of selected miRNAs. DESeq2 analysis of the HTS data was conducted by comparing one organ or early developmental stage (*n* = 2) to all other samples (*n* = 14). The results (Table S7) suggested that several miRNAs have higher or lower expression in one organ or early developmental stages compared to all other samples. The suggested miRNAs with an increased expression (log_2_ fold change > 3.0) in a particular organ or early developmental stage compared to expression in all others are given in Table 2 and Table 3, respectively. The numbers of such miRNAs were 9 in the brain, 5 in muscle, 8 in gill, 15 in the liver, 3 in the spleen, 13 in embryos, and 22 in larvae. However, our DESeq2 analysis did not suggest any enrichment of miRNAs in the lumpfish head kidney.

**Figure 5:**
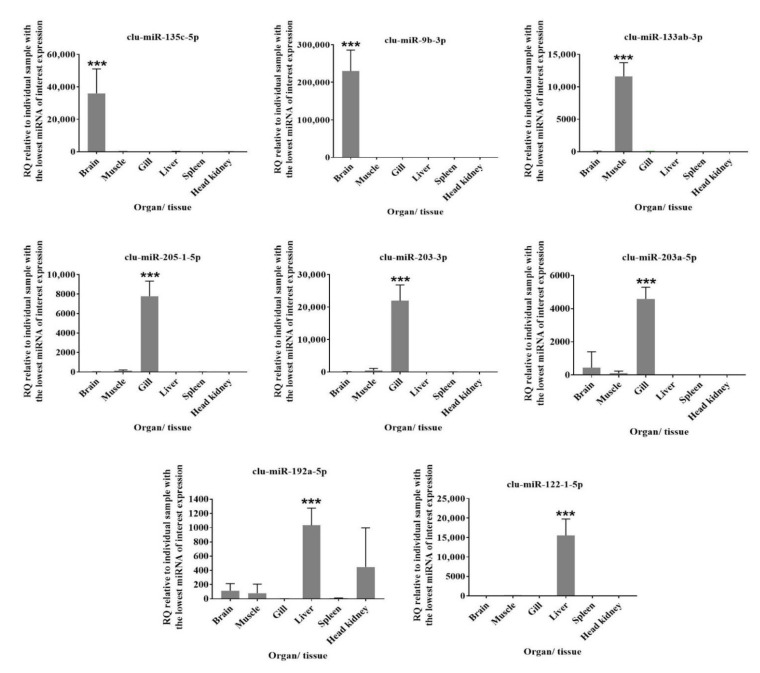
Verification of tissue-specific expression of conserved miRNAs. RT-qPCR results show the relative expression of eight miRNAs (clu-miR-135c-5p, clu-miR-9b-3p, clu-miR-133ab-3p, clu-miR-205-1-5b, clu-miR-203-3p, clu-miR-203a-5p, clu-miR-192a-5p, clu-miR-122-1-5p ) across lumpfish organs (brain, muscle, gill, liver, spleen, and head kidney). Number of replicates for tissue samples were five (*n* = 5) except brain (*n* = 4) and muscle (*n* = 4). RQ: relative quantity normalized to clu-miR-25-3p and clu-miR-17-1-5p and calibrated to the individual sample with the lowest miRNA of interest expression. *** on the top of a particular sample indicates that the expression of the particular miRNA is significantly higher when compared to others by one-way ANOVA (*p* < 0.001).

RT-qPCR was applied to verify the findings from the DESeq2 analysis in a few selected miRNAs. The two conserved mature miRNAs clu-mir-25-3p and clu-mir-17-5p, shown as suitable reference genes in other teleosts [20,55], revealed stable expression across all samples in this study (mean Ct values were 22.8 ± 0.9 (SD), 22.4 ± 1.1 (SD), respectively) and were, consequently, used as reference genes in the RT-qPCR analysis. Eight miRNAs known to be highly expressed in certain organs [19,20] were selected for RT-qPCR (Table 1). These selected miRNAs showed significantly increased expression levels in the expected tissue/organs (Figure 5) that align with the literature [19,20]. For instance, clu-miR-135c-5p and clu-miR-9b-3p expression was significantly higher in brain, clu-miR-205b-5b, clu-miR-203a-3p, and clu-miR-203b expression was significantly higher in gill, clu-miR-133-3p expression was significantly higher in muscle, and clu-miR-122-5p and clu-miR-192a-5p expression was significantly higher in liver compared with other tissue/organs. These qPCR results agreed with the DESeq2 results for six organ samples, while the increases observed for clu-miR-135c-5p and clu-miR-9b-3p in the brain were similar in the DESeq2 analysis but not significant. However, we utilized four or five biological replicates for the RT-qPCR analysis, whereas two were used in the DESeq2 analysis. Additionally, the significance levels were adjusted according to a large number of tests in the DESeq2 analysis. This could explain why the increases did not reach the significant thresholds in the DESeq2 analysis for these two miRNAs. 

## 4. Discussion

miRNAs play a significant role in embryonic development, determination of cell fate, and control of cell proliferation, differentiation, and death. Their dysregulation has a significant impact on critical cellular pathways and is linked to a variety of diseases [11,13,16,48]. A species-specific and well-characterized miRNAome generated from small RNA sequencing of different developmental stages is required to study miRNA expression by analysis of HTS data. Characterization of miRNAs in multiple organs and developmental stages in a new aquaculture species like lumpfish will also facilitate further studies to determine their role in development, whether they regulate organ developmental stage-specific functions, immune responses to infectious diseases, and disease progression. Therefore, this study was undertaken to define and characterize miRNAs expressed in the brain, muscle, gill, liver, spleen, and head kidney of adult lumpfish, as well as the two developmental stages, embryos and larvae. Together this resulted in a miRNAome consisting of 443 unique mature miRNAs that were used as lumpfish miRNA reference for analysis of HTS data and primer design (RT-qPCR analysis) of single miRNAs. 

The expression of different miRNAs within an organ or developmental stage would reveal which ones were highly abundant and likely to have essential regulatory functions. Comparisons between adult organs and early developmental stages could further reveal the highly expressed ones in a few or single organs. We applied DESeq2 analysis to demonstrate that the miRNAome worked well as a reference in such HTS analysis. However, as there were two biological replicates of each adult organ (or early developmental stages) compared to all other HTS samples (*n* = 14) in these analyses, we report them as suggestive expression differences. Ideally, there should be three or more biological replicates in each group compared in such analysis. However, we did choose a rather conservative log_2_FC (3 or more) to suggest them as differently expressed between organs (Table S7), and some of the miRNAs increased in particular organs were also supported in the additional RT-qPCR analysis (Figure 5).

Our analysis identified that 10 mature miRNAs were highly abundant and among the top 20 enriched miRNAs within all six organs (five were also among the top 20 enriched in the early developmental stages). These 10 mature miRNAs (clu-let-7a-3-5p, clu-let-7e-5p, clu-let-7g-5p, clu-miR-21a-5p, clu-miR-22ab-3p, clu-miR-26-1-5p, clu-miR-100-2-5p, clu-miR-126-3p, clu-miR-143-3p, and clu-miR-146a-5p) are conserved miRNA families discovered in the majority of vertebrates in miRBase [19,20,58]. Their high expression within all adult organs could suggest that these miRNAs play a critical role in lumpfish cellular homeostasis. Still, as they are highly abundant in all adult organs, they are not likely to regulate organ-specific functions.

The brain receives information from sense organs that monitor conditions both within and around the fish. In the brain, the immune cells and the central nervous system interactions allow the immune system to fight against infection and enable the nervous system to regulate immune functioning [59,60]. Any change in these interaction pathways can cause many pathological conditions attributed to organ dysfunction [59,60]. However, miRNAs are critical brain development and function regulators, such as neuronal activity [11,61]. Our miRDeep2 analysis identified 340 conserved mature miRNAs in the lumpfish brain. Among highly enriched in the brain are clu-miR-9-2-5p and clu-miR-7-3-5p. These two miRNAs do not have similar high relative expression levels within any other adult organs but are similarly enriched in the two early developmental stages, indicating they could be important in developing neural tissue in lumpfish (Table S6). A similar enrichment pattern of miR-9-5p is seen in Atlantic salmon, cod, halibut, three-spined stickleback, and zebrafish brain [19,20,22,28]. Enrichment of miR-7 in the brain is also observed across vertebrates [62]. Several studies have shown that these two miRNAs are crucial for brain development in zebrafish and other vertebrates [63,64,65], and it is likely that clu-miR-9-2-5p and clu-miR-7-3-5p may have similar functions in lumpfish. The DESeq2 analysis also suggested that clu-miR-128-2-3p, clu-miR-153c-3p, clu-miR-212b-1-5p, and clu-miR-338-1-3p were more than 10-times higher expressed in the brain than other organs (Table S7a). Similar findings were observed in Atlantic cod, three-spined stickleback, and zebrafish [19,66]. In higher vertebrates, miR-128 controls neural motor behaviours by regulating the expression of various ion channels [67]. The three other miRNAs have also all been reported as having important brain functions in higher vertebrates [68,69,70].

Fish muscles are the major edible parts worldwide, determining the nutritional and the market value. The teleost muscle is also an immunologically active organ, playing an important role against pathogens [71]. MicroRNAs are established modulators of muscle cell proliferation, differentiation, regeneration, and diseases [72]. Our miRDeep2 analysis identified 328 conserved mature miRNAs in lumpfish muscle. The muscle-specific top enriched miRNAs were clu-miR-1-1-3p and clu-miR-133ab-3p (present in other organs but much less abundant). Similar to our study, miR-133 and miR-1 were enriched in zebrafish, Atlantic salmon, and cod, suggesting the maintenance of muscle-specific miRNAs expression and function [18,19,20,73]. For example, miR-133 is one of the foremost studied and best-characterized miRNAs in vertebrates. It is required for proper skeletal and cardiac muscle development and function in mammals and fish [74,75]. On the other hand, miR-1 is a conserved miRNA in the muscle tissue that plays a crucial role in maintaining muscle integrity [76]. 

Because of direct exposure to the water, teleost gills are the main mucosal surfaces for the entrance of pathogens, which trigger an immune response [77]. miRNAs are important regulators of immune response to those infections in the gills of fish [20,78,79]. However, our DESEq2 analysis was on apparently healthy organs and suggested the enrichment of clu-miR-200 and clu-miR-203 family members and clu-miR-205-1-3p, clu-miR-375-1-3p, clu-miR-31-5p, and clu-miR-1788-3p in lumpfish gill. RT-qPCR results confirmed the enrichment of clu-miR-203-3p, clu-miR-203a-5p, and clu-miR-205-1-5p. Some of these miRNAs, such as miR-200, miR-205, and miR-375, were enriched in cod gill as well [20], while miR-200, miR-203, and miR-205 were enriched in gills of tilapia [80]. One of these miRNAs, miR-200, has been shown as important to gill function in cell studies of fish [81]. However, no study has been conducted to decipher the gill-associated role of the remaining five lumpfish miRNAs suggested as differentially expressed in teleost gill. 

The liver is involved in various vital functions in controlling biochemical processes, including detoxification and metabolism [82]. miRNAs are essential for regulating liver development and functions, and alterations in intrahepatic miRNA networks have been associated with liver disease in humans [83]. They are also associated with hepatic lipid metabolism in Atlantic salmon [84]. Our miRDeep analysis identified 288 conserved mature miRNAs in the lumpfish liver (Table S6). 

Four of the top 20 enriched miRNAs in liver—clu-miR-122-1-5p, clu-miR-152ab-3p, clu-miR-192a-5p and clu-miR-nov5-5p—did not show similar enrichment in any other adult organs (Figure 3). DESEq2 analysis also suggested these as having significantly increased expression in the liver, and this was confirmed by RT-qPCR for clu-miR-122-1-5p and clu-miR-192a-5p (Figure 5). This finding is similar to other teleosts and mammals [20,85,86]. miR-122 is the most abundant miRNA in the liver of many species. In mammals, miR-122 is studied extensively and is known to be involved in lipid metabolism [85]. Furthermore, miR-192 is involved in cell growth, lipid synthesis, and apoptosis [87] and having such roles also aligns with this miRNA being among the top 20 miRNAs expressed in the early developmental stage samples (Figure 4). Dysregulation of miR-152 is associated with liver disease in higher vertebrates indicating they are important hepatic miRNAs [84,88]. Based on the high conservation of these miRNAs among vertebrates (miRBase 22.1) [58] and with a similar enrichment pattern observed in lumpfish, we could assume they also play a similar liver-specific role in lumpfish.

As the body’s primary blood filter, the spleen plays a major role in detecting cell damage during infection [89]. The spleen is the home of different types of immune cells that trigger different immune responses [89,90,91]. Splenic miRNAs have been identified to modulate immune responses during diseases in humans, mice, chickens, dogs, and fishes [92,93,94,95,96,97,98,99]. Our miRDeep analysis identified 268 conserved mature miRNAs in the lumpfish spleen. One mature miRNA, clu-miR-451a-5p, was only among the top 20 enriched miRNAs in the spleen, and this particular miRNA has been shown to regulate erythroid maturation in zebrafish [100]. Furthermore, our DESeq2 analysis suggested the enrichment of clu-miR-2187b-5p and clu-miR-460-5p in the lumpfish spleen. These two miRNAs are also enriched in Atlantic salmon and cod spleen [19,20], but their particular function in the spleen has not yet been investigated.

The anteriormost part of the kidney in the teleost is referred to as the head kidney. It is predominantly a lymphoid compartment. The head kidney is an essential hematopoietic organ and serves as a secondary lymphoid organ, a lymph node analog, vital in inducing and elaborating immune responses [90,91]. Assessing changes in the expression of miRNAs in the head kidney could provide more comprehensive insight into the immune response to infection. Our miRDeep2 analysis identified 289 conserved mature miRNAs in the lumpfish head kidney. Our DESeq2 analysis did not suggest any enrichment of miRNA in the head kidney. 

The embryos and larvae samples did reveal several miRNAs suggested as early developmental stage enriched. Notably, the miR-430 family was suggested as enriched by the DESeq2 analysis. These are known as highly expressed in early development, and among suggested functions is maternal RNA clearance during early embryogenesis in zebrafish [101,102]. Another miRNA that was highly enriched and expressed only in the early developmental stages was clu-miR-217b-5p. This miRNA, as well as mature miRNAs from miR-124, miR-184, and miR-216 families that were also enriched in the lumpfish early developmental stages, have all been shown as important in zebrafish development [73].

## 5. Conclusions

In conclusion, this study represents the first characterization of a lumpfish miRNA transcriptome produced by independent analysis of small RNA sequences from several adult organs and early developmental stages. We identified 391 conserved and eight novel miRNA precursor sequences, which account for 443 unique mature miRNAs. Our results demonstrate that most of the lumpfish miRNAs are highly conserved with highly similar precursor sequences to those observed in other teleosts. Many miRNAs also appear to have similar tissue-specific expression patterns as in other vertebrates. Thus the miRNAs profile of lumpfish suggested a similar organ-specific expression pattern as other vertebrates. It is possible that these conserved miRNAs are regulating essential and conserved genes in vertebrates. Furthermore, the identification and characterization of lumpfish-specific novel miRNAs repertoire in this study will be crucial for further functional studies of the novel miRNAs in this species.

## Figures and Tables

**Table 1:** Primers used in qPCR analysis of mature miRNAs.

miRNAs	Primer Sequences (5′ to 3′)
clu-miR-25-3p	CATTGCACTTGTCTCGGTCTGA
clu-miR-17-1-5p	CAAAGTGCTTACAGTGCAGGTA
clu-miR-122-1-5p	TGGAGTGTGACAATGGTGTTTG
clu-miR-133ab-3p	TTTGGTCCCCTTCAACCAGCTGT
clu-miR-205-1-5p	TCCTTCATTCCACCGGAGTCTG
clu-miR-135c-5p	TATGGCTTTTTATTCCTATGTG
clu-miR-203-3p	GTGAAATGTTTAGGACCACTTG
clu-miR-203a-5p	AGTGGTTCTCAACAGTTCAACA
clu-miR-192a-5p	ATGACCTATGAATTGACAGCCA
clu-miR-9b-3p	TAAAGCTAGAGAACCGAATGTA

**Table 2:** Mature miRNAs suggested as highly expressed in one organ compared to others.

Organ ^1^	miRNAs ^2^	Log_2_FC ^3^
Brain	clu-miR-31-3p	6.14
Brain	clu-miR-153c-3p	5.96
Brain	clu-miR-153a-3p	5.33
Brain	clu-miR-1788-5p	4.91
Brain	clu-miR-212b-1-5p	4.10
Brain	clu-miR-212b-1-3p	3.14
Brain	clu-miR-128-2-3p	3.49
Brain	clu-miR-338-1-3p	3.40
Brain	clu-miR-132-1-5p	3.08
Muscle	clu-miR-133b-3p	6.08
Muscle	clu-miR-133ab-3p	5.45
Muscle	clu-miR-1-1-3p	5.23
Muscle	clu-miR-1-3-5p	3.56
Gill	clu-miR-31-5p	6.91
Gill	clu-miR-1788-3p	6.21
Gill	clu-miR-203-3p	5.13
Gill	clu-miR-203a-5p	4.61
Gill	clu-miR-375-1-3p	4.82
Gill	clu-miR-205-1-3p	4.16
Gill	clu-miR-200b-3p	3.8
Gill	clu-miR-200b-5p	3.36
Liver	clu-miR-122-1-5p	8.23
Liver	clu-miR-122-1-3p	7.65
Liver	clu-miR-nov3-3p	6.78
Liver	clu-miR-nov3-5p	4.68
Liver	clu-miR-nov1-5p	5.58
Liver	clu-miR-101b-3p	4.83
Liver	clu-miR-101b-5p	4.41
Liver	clu-miR-722-3p	4.71
Liver	clu-miR-722-5p	4.38
Liver	clu-miR-92b-3p	4.04
Liver	clu-miR-92b-5p	3.91
Liver	clu-miR-192a-5p	3.75
Liver	clu-miR-94a-5p	3.43
Liver	clu-miR-152ab-3p	3.37
Liver	clu-miR-nov5-5p	3.36
Spleen	clu-miR-2187b-5p	5.10
Spleen	clu-miR-2187b-3p	3.47
Spleen	clu-miR-460-5p	3.27

^1^ Organ samples were obtained from adult lumpfish. ^2^ The names are in a few cases with different lettered/numbered suffixes than in miRBase as several mature family members are identical. The miRNAs in the table are grouped in families, and the family member with the highest FC is used to list families in descending order. ^3^ Log_2_-transformed fold-change (FC) as determined by DESeq2 analysis.

**Table 3:** Mature miRNAs suggested as highly expressed in embryos or larvae.

Embryos/Larvae ^1^	miRNAs ^2^	Log_2_FC ^3^
Embryos	clu-miR-430b-5-5p	5.61
Embryos	clu-miR-430b-4-3p	4.51
Embryos	clu-miR-430b-1-3p	4.37
Embryos	clu-miR-190b-5p	5.45
Embryos	clu-miR-726-5p	4.91
Embryos	clu-miR-184ab-2-3p	4.77
Embryos	clu-miR-184ab-3p	4.77
Embryos	clu-miR-301b-5p	4.73
Embryos	clu-miR-301b-1-5p	4.40
Embryos	clu-miR-124-1-5p	4.40
Embryos	clu-miR-217b-5p	4.23
Embryos	clu-miR-217a-5p	4.13
Embryos	clu-miR-216a-1-5p	4.20
Larvae	clu-miR-124-1-5p	4.41
Larvae	clu-miR-130-1-5p	3.15
Larvae	clu-miR-130-6-5p	3.62
Larvae	clu-miR-183-5p	4.09
Larvae	clu-miR-184ab-2-3p	4.71
Larvae	clu-miR-184ab-3p	4.71
Larvae	clu-miR-190b-5p	5.44
Larvae	clu-miR-194b-3p	3.46
Larvae	clu-miR-196a-1-5p	3.95
Larvae	clu-miR-216a-1-5p	4.00
Larvae	clu-miR-217a-5p	4.11
Larvae	clu-miR-217b-5p	4.11
Larvae	clu-miR-301b-1-5p	4.38
Larvae	clu-miR-301b-3p	3.44
Larvae	clu-miR-301b-5p	4.70
Larvae	clu-miR-430a-12-3p	3.97
Larvae	clu-miR-430a-3-3p	3.97
Larvae	clu-miR-430b-1-3p	4.28
Larvae	clu-miR-430b-4-3p	4.40
Larvae	clu-miR-430b-5-5p	5.41
Larvae	clu-miR-459-3p	4.01
Larvae	clu-miR-726-5p	4.64

^1^ Lumpfish embryos were obtained at 300 degree days, and lumpfish larvae were obtained after one-week post-hatch. ^2^ The names are in a few cases with different lettered/numbered suffixes than in miRBase as several mature family members are identical. The miRNAs in the table are grouped in families, and the family member with the highest FC is used to list families in descending order. ^3^ Log_2_-transformed fold-change (FC) as determined by DESeq2 analysis.

## Data Availability

All raw HTS results were submitted to NCBI with bio project accession number PRJNA679415. The individual SRA accession numbers are given in Table S2.

## Supplementary Materials

The following are available online at https://www.mdpi.com/article/10.3390/biology11010130/s1, Table S1: Concentration and quality of RNA samples, Table S2: Descriptive data from small RNA sequenced samples, Table S3: All evolutionarily conserved and novel lumpfish miRNA genes discovered in different organs and developmental stages, File S4: Unique mature miRNAs, Table S5, Normalized read counts of individual mature miRNAs from sequencing data in each sample sequenced, Table S6: Relative distribution of miRNAs within organs and within developmental stages, Table S7: Mature miRNAs suggested as differentially expressed by DESeq2 analysis, Table S8: miRNAs analyzed by qPCR.
